# Magnesium as a diagnostic marker of cancer

**DOI:** 10.1186/1897-4287-13-S2-A6

**Published:** 2015-11-26

**Authors:** Wojciech Marciniak, Magdalena Muszyńska, Katarzyna Jaworska-Bieniek, Katarzyna Kaczmarek, Grzegorz Sukiennicki, Marcin Lener, Katarzyna Durda, Tomasz Huzarski, Tomasz Byrski, Jacek Gronwald, Oleg Oszurek, Cezary Cybulski, Tadeusz Dębniak, Antoni Morawski, Anna Jakubowska, Jan Lubiński

**Affiliations:** 1 Department of Genetics and Pathology, International Hereditary Cancer Center, Pomeranian Medical University, Szczecin, Poland; 2 Read - Gene, S.A., Grzepnica, Poland

## 

Magnesium plays a key role in many essential cellular processes. It is recognized that magnesium deficiency may lead to many disorders like cardiovascular disease, diabetes mellitus, hypertension, myocardial infarction or even cancer. Connection between magnesium concentration in body fluids and cancer occurrence still becomes unclear. Magnesium ions are crucial cofactors for enzymes involved DNA repairing. Thereby magnesium deficiency may result in genomic instability and consequently cause cancer.

The aim of our study was to investigate if serum magnesium concentration may be useful biomarker for early detection of prostate and laryngeal cancer and also if serum Mg concentration may be marker of selection for CT of the lung, laryngeal examination, PSA levels or prostate biopsy. Independently we have investigated a prospective study where our aim was to investigate if the serum Mg concentration is a marker of increased breast cancer risk. We performed retrospective investigation in laryngeal cancer patients (n = 123) and prostate cancer groups (n = 166). Each group was compared with healthy controls group, which consists of equal to cases amount of controls. Controls and cases were matched by age, gender, negative supplementation status, negative status of hormonal contraceptives and smoking status. In the prospective study (sera collected before cancer diagnosis) Mg concentration was determined in BRCA (-) women and compared to controls. Mg serum levels (<17000 µg/l and >20800 µg/l) are associated with 2-fold higher occurrence of laryngeal cancers. Magnesium serum level is not a useful marker for selection of the patients for prostate cancer screening. Finally, magnesium concentration may be a marker for breast cancer risk in BRCA (-) patients but further investigation is needed.

